# Biodistribution of charged 17.1A photoimmunoconjugates in a murine model of hepatic metastasis of colorectal cancer

**DOI:** 10.1054/bjoc.2000.1486

**Published:** 2000-11-22

**Authors:** M R Hamblin, M Del Governatore, I Rizvi, T Hasan

**Affiliations:** Wellman Laboratories of Photomedicine, Massachusetts General Hospital, and Department of Dermatology, Harvard Medical School, Boston, MA, 02114, USA; Department of Chirurgia III, Ospedale Policlinico S. Orsola, Via Massarenti 9, Bologna, 40100, Italy

**Keywords:** photodynamic therapy, photoimmunotherapy, monoclonal antibody, photosensitizer, polylysine, intraperitoneal PDT

## Abstract

Optimizing photodynamic therapy involves attempting to increase both the absolute tumour content of photosensitizer and the selectivity between tumour and surrounding normal tissue. One reason why photodynamic therapy has not been considered suitable for treatment of metastatic tumours in the liver, is the poor selectivity of conventional photosensitizers for tumour compared to normal liver. This report details an alternative approach to increasing this selectivity by the use of antibody-targeted photosensitizers (or photoimmunoconjugates) to target intrahepatic tumours caused by human colorectal cancer cells in the nude mouse, and explores the role of molecular charge on the tumour-targeting efficiency of macromolecules. The murine monoclonal antibody 17.1A (which recognizes an antigen expressed on HT 29 cells) was used to prepare site-specific photoimmunoconjugates with the photosensitizer chlorine6. The conjugates had either a predominant cationic or anionic charge and were injected i.v. into tumour-bearing mice. Biodistribution 3 or 24 h later was measured by extraction of tissue samples and quantitation of chlorine6 content by fluorescence spectroscopy. The photoimmunoconjugates were compared to the polylysine conjugates in an attempt to define the effect of molecular charge as well as antibody targeting. The anionic 17.1A conjugate delivered more than twice as much photosensitizer to the tumour at 3 h than other species (5 times more than the cationic 17.1A conjugate) and had a tumour:normal liver ratio of 2.5. Tumour-to-liver ratios were greater than one for most compounds at 3 h but declined at 24 h. Tumour-to-skin ratios were high (> 38) for all conjugates but not for free chlorine6. Cationic species had a high uptake in the lungs compared to anionic species. The photoimmunoconjugates show an advantage over literature reports of other photosensitizers, which can result in tumour:normal liver ratios of less than 1. © 2000 Cancer Research Campaign http://www.bjcancer.com


*British Journal of Cancer* (2000) **83**(11), 1544–1551
© 2000 Cancer Research Campaign
doi: 10.1054/ bjoc.2000.1486, available online at http://www.idealibrary.com on IDEAL®

http://www.bjcancer.com

### Biodistribution of charged 17.1A photoimmunoconjugates in a murine model of hepatic metastasis of colorectal cancer

**MR Hamblin[1], M Del Governatore[1,2], I Rizvi[1] and T Hasan[1]**

[1]Wellman Laboratories of Photomedicine, Massachusetts General Hospital, and Department of Dermatology, Harvard Medical School, Boston, MA 02114, USA;
[2]Department of Chirurgia III, Ospedale Policlinico S. Orsola, Via Massarenti 9, Bologna 40100, Italy

Summary Optimizing photodynamic therapy involves attempting to increase both the absolute tumour content of photosensitizer and the selectivity between tumour and surrounding normal tissue. One reason why photodynamic therapy has not been considered suitable for treatment of metastatic tumours in the liver, is the poor selectivity of conventional photosensitizers for tumour compared to normal liver. This report details an alternative approach to increasing this selectivity by the use of antibody-targeted photosensitizers (or photoimmunoconjugates) to target intrahepatic tumours caused by human colorectal cancer cells in the nude mouse, and explores the role of molecular charge on the tumour-targeting efficiency of macromolecules. The murine monoclonal antibody 17.1A (which recognizes an antigen expressed on HT 29 cells) was used to prepare site-specific photoimmunoconjugates with the photosensitizer chlorine6. The conjugates had either a predominant cationic or anionic charge and were injected i.v. into tumour-bearing mice. Biodistribution 3 or 24 h later was measured by extraction of tissue samples and quantitation of chlorine6 content by fluorescence spectroscopy. The photoimmunoconjugates were compared to the polylysine conjugates in an attempt to define the effect of molecular charge as well as antibody targeting. The anionic 17.1A conjugate delivered more than twice as much photosensitizer to the tumour at 3 h than other species (5 times more than the cationic 17.1A conjugate) and had a tumour:normal liver ratio of 2.5. Tumour-to-liver ratios were greater than one for most compounds at 3 h but declined at 24 h. Tumour-to-skin ratios were high (> 38) for all conjugates but not for free chlorine6. Cationic species had a high uptake in the lungs compared to anionic species. The photoimmunoconjugates show an advantage over literature reports of other photosensitizers, which can result in tumour:normal liver ratios of less than 1. © 2000 Cancer Research Campaign http://www.bjcancer.com

**Keywords**: photodynamic therapy; photoimmunotherapy; monoclonal antibody; photosensitizer; polylysine; intraperitoneal PDT

A major cause of death from colorectal cancer is liver metastasis, which at present has a bleak prognosis and is in urgent need of novel therapies (Van Cutsem, 1996), one of which may be photodynamic therapy (PDT) (Dougherty et al, 1998). PDT has not previously been much used to treat liver tumours for two reasons. Firstly, it is known that normal liver tissue accumulates large amounts of conventional photosensitizers (PS) (Woodburn et al, 1992). In the case of the only PS with clinical approval Photofrin® (Dougherty et al, 1998), this fact can lead to the occurrence of reverse selectivity, where the normal liver actually has higher concentrations of PS than the tumour (Van Hillegersberg et al, 1992). Secondly, the transmission of light through the highly pigmented liver tissue is relatively poor compared to other tissue types (van Hillegersberg et al, 1993). The latter drawback may be overcome by selecting a PS, which absorbs further in the red and using interstitial illumination via a fibre inserted into the tumour. The first drawback may be overcome by seeking ways to increase the selectivity of the PS for colorectal cancer cells at the expense of normal liver parenchymal cells. One way of accomplishing this is to attach the PS to a monoclonal antibody (Mab) which recognizes tumour specific antigens expressed on the surface of tumour cells (Mew et al, 1983). Our laboratory has explored this approach to experimental treatment of peritoneal dissemination of ovarian cancer (Goff et al, 1992, 1994, 1996), and other workers have applied this clinically in ovarian cancer treatment (Schmidt et al, 1992a, 1992b). The Mab construct is known as a photoimmunoconjugate (PIC), and we have reported (Hamblin et al, 1996; Duska et al, 1997) on a method of preparing these PICs in a site-specific manner using poly-L-lysine linkers to attach the PS chlorin$_{e6}$ ($c_{e6}$) and which allows them to be prepared with predominant cationic or anionic charges.

17.1A is a murine antibody (Gottlinger et al, 1986) that recognizes the epithelial membrane antigen (a homophilic cell–cell adhesion molecule known as Ep-CAM); this antigen is overexpressed on many cancers of the gastrointestinal tract (Litvinov et al, 1994). 17.1A has been used in experimental clinical studies to treat human colorectal cancer, both in an unconjugated form to induce antibody-dependent cellular cytotoxicity (Riethmuller et al, 1994) and as radioimmunoconjugates to target radioisotopes to residual tumour (including liver metastases) (Meredith et al, 1995). In a previous report we detailed the preparation of PICs between 17.1A and $c_{e6}$ and which bore either polycationic or polyanionic charges (Del Governatore et al, 2000a) (Figure 1). Both these charged PICs preserved antigen-binding capacity, and showed selective uptake and phototoxicity towards target HT29 cells.

PDT or photoimmunotherapy might have a role to play in treating liver tumours which are not amenable to surgery, but

*Received 3 April 2000*
*Revised 24 July 2000*
*Accepted 1 August 2000*

*Correspondence to*: T Hasan

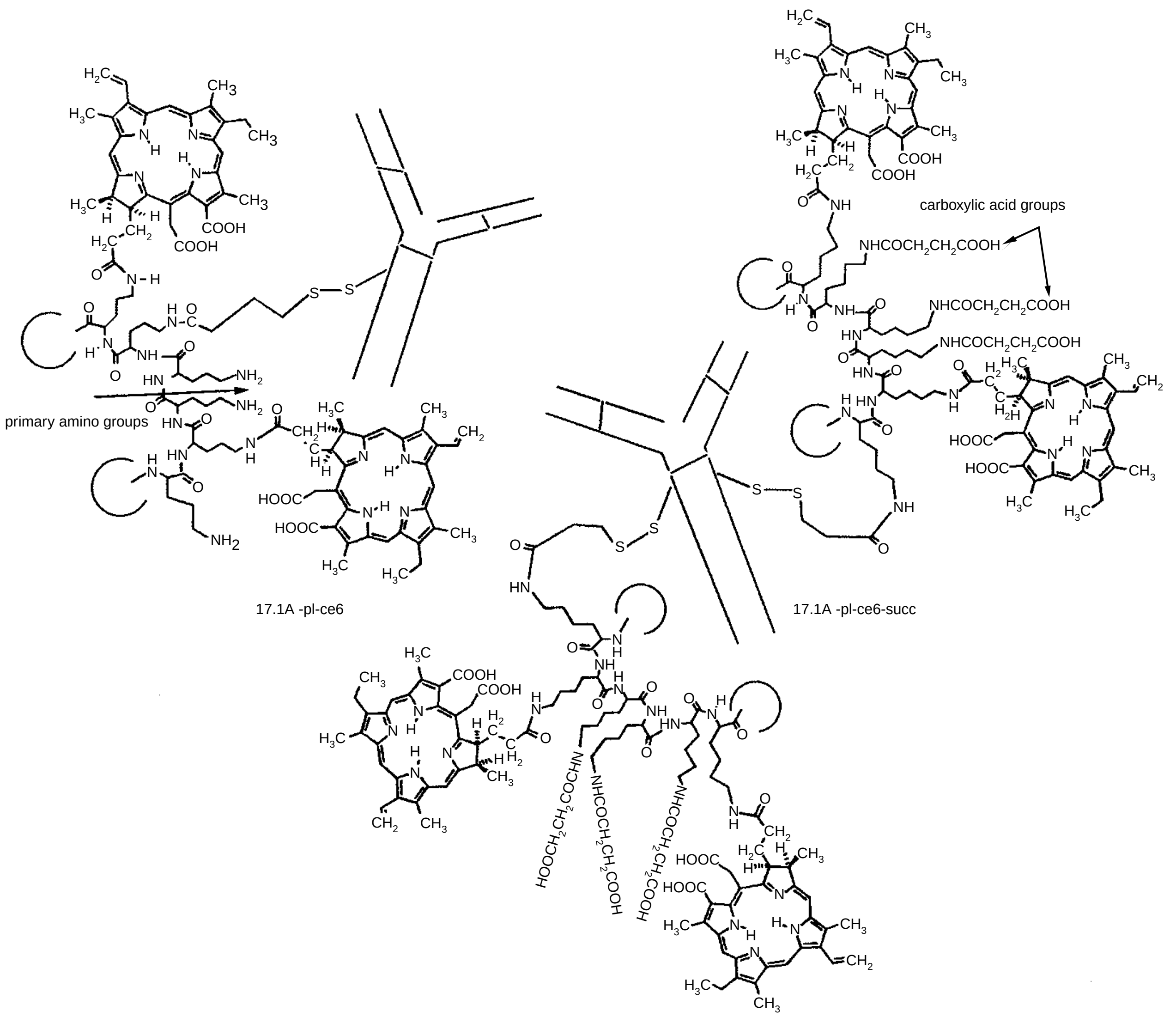

**Figure 1** Structural representation of the PICs. 17.1A-pl-$c_{e6}$ has primary amino groups, which give it a polycationic charge, while 17.1A-pl-$c_{e6}$-succ has carboxylic groups that give it a polyanionic charge. 17.1A-pl-$c_{e6}$ contains 1 pl-$c_{e6}$ chain per Mab, while 17.1A-pl-$c_{e6}$-succ contains 2

which nevertheless are localized within the liver. It may be possible to deliver the PS or PIC in a locoregional approach via the hepatic artery (Nishiwaki et al, 1989; Rovers et al, 1999). The utility of this approach can only be established if selective delivery to tumour can be demonstrated in vivo. As a first step towards this goal the present study explores the biodistribution of these cationic and anionic PICs in a nude mouse model of hepatic metastases of human colorectal cancer. We previously showed (Duska et al, 1997) that for i.p. delivery to i.p. tumours a PIC with a cationic charge performed better than one with an anionic charge. However, the effect of charge on the biodistribution of intravenously delivered immunoconjugates is uncertain, but considering the short serum half-life of cationic macromolecules (Pardridge et al, 1998), we hypothesized that the anionic 17.1A PIC would perform more efficiently in vivo. The experiments were designed to study the biodistribution of cationic and anionic 17.1A PICs, the component cationic and anionic pl-$c_{e6}$ conjugates and the free PS $c_{e6}$, in order to assess the effect of both charge and antibody recognition on the selectivity for tumour over normal liver. Two time points (3 hours and 24 hours) after administration were employed in order to assess the balance between absolute amounts of PS in the tumour, and tumour-to-normal liver ratio. This study should provide data for choosing parameters suitable for intraperitoneal photoimmunotherapy of liver metastasis of human colorectal cancer in the nude mouse.

### MATERIALS AND METHODS

### Mice

All experiments were carried out with the approval of the Subcommittee on Research Animal Care of Massachusetts General Hospital and were in accord with the NIH Health Guide for the Care and Use of Laboratory Animals. Female Swiss nude mice (Cox Breeding Laboratories, Cambridge, MA) (2–3 weeks old, weighing 20–25 g) were kept in a barrier room under permanent sterile conditions to avoid any infections and had continual access to food and water, which was taken ad libitum. Throughout the experiment mice were housed in laminar flow racks under specific pathogen-free conditions, and were monitored daily for general health status.

### Cell line and monoclonal antibody

HT29 tumour cell line derived from a human colorectal adenocarcinoma, was a generous gift from Dr K Tanabe (Massachusetts General Hospital, Boston, MA). Cells were grown in DMEM/F12 (50/50 MIX) containing 15 mM HEPES and l-glutamine and were supplemented with 10% heat-inactivated FBS (Whittaker Bioproduct, Walkersville, MD), 100 units $ml^{-1}$ penicillin and 100 µg $ml^{-1}$ streptomycin, and maintained in an incubator at 37˚C in an atmosphere of 5% $CO_2$. 17.1A murine monoclonal antibody was a kind gift from Centacor (Malvern, PA).

### Preparation and characterization of PICs

This has been described previously (Del Governatore et al, 2000a). Briefly 17.1A IgG was partially reduced with mercaptoethylamine hydrochloride and reacted with one of two poly-L-lysine $c_{e6}$ conjugates which had been derivatized with a heterobifunctional reagent bearing a pyridyldithiopropionamide group in order to form a disulphide bond between the IgG hinge sulphydryl group and the pl-$c_{e6}$ conjugate. The two PICs had opposite charges: the anionic 17.1A-pl-$c_{e6}$-succ had a loading of 8–9 $c_{e6}$ molecules (2 pl-$c_{e6}$-succ chains) per Mab, while the cationic 17.1A-pl-$c_{e6}$ had a loading of 4–5 $c_{e6}$ molecules (1 pl-$c_{e6}$ chain) per Mab. Their structures are shown in Figure 1. Their immunoreactivity was demonstrated by two colour direct/indirect immunofluorescence and ELISA assays (Del Governatore et al, 2000a). Also available were identical constituent polylysine conjugates, pl-$c_{e6}$ and pl-$c_{e6}$-succ, which had identical loadings of 5 $c_{e6}$ per polylysine chain of 225 lysine residues.

### Animal model

A xenograft model for liver metastases of colorectal cancer was developed in our laboratory and utilized for the experiment. Mice were anaesthetized by inhalation of Metofane (Pitman-Moore Inc, Mundelein, IL); 2 ml liquid vaporized in a 500 ml closed container. Under aseptic condition mice were placed in a supine position and a 1 cm left median incision (starting from sub-costal region) was made through the skin and the peritoneum to expose the left lateral lobe of the liver. That lobe was lifted out from the abdominal cavity and secured in place by positioning a sterile cotton-tipped stick inferior to the lobe. HT29 cells ($5 \times 10^6$) in 50 µl of sterile DMEM/F12 were injected between the upper surface of the lobe parenchyma and the liver capsule using a 30-gauge needle in each mouse and after this the lobe was returned into the peritoneal cavity. The puncture wound in the capsule was sterilized with 100 µl povidone iodine 10% (Clinidine Solution, Clinipad Corp., Guilford, CT). The peritoneum and the abdominal wall were closed with sterile Ethilon 4-0 monofilament nylon sutures (Ethicon Inc, Somerville, NJ) and the mice were monitored and kept warm until they recovered completely from the procedure.

### Biodistribution

Experiments took place 9 days after tumour cell injection. Five different $c_{e6}$-based photosensitizing agents were used: free $c_{e6}$, pl-$c_{e6}$, pl-$c_{e6}$-succ, 17.1A-pl-$c_{e6}$, and 17.1A-pl-$c_{e6}$-succ. The injected dose of 0.25 mg $c_{e6}$ equivalent / kg body weight (approximately 8.3 nmol $mouse^{-1}$) was the same for all the compounds tested and involved the injection (lasting about 20 seconds) of 40 µl of a 200 µM $c_{e6}$ equivalent solution in sterile PBS per mouse in the tail vein with mice under anaesthesia (Metofane, 2 ml vapour in closed system). For the anionic PIC this involved the injection of approximately 145 µg 17.1A IgG per mouse, while for the cationic PIC the dose contained 275 µg 17.1A IgG. At time points 3 h and 24 h after injection (*n* = 6–10 mice/time point) animals were sacrificed by $CO_2$ inhalation. At necropsy the normal liver, tumour, blood, skin, muscle, kidney, spleen, small intestine, stomach, bladder, lung, and heart were harvested. Wet tissue samples (about 100 mg) were weighed immediately after resection and frozen at –70˚C. For extraction of the photosensitizer, the tissues were thawed and homogenized (homogenizer model PT 10/35; Brinkman Instruments, Westbury, NY) in 2 ml 1 M NaOH/0.2% SDS for 30 seconds and centrifuged at 1000 ***g*** (Sorvall RC-5B, refrigerated superspeed centrifuge; Dupont Sorvall, Newtown, CT) at 20˚C for 15 min and the supernatant was collected by aspiration. Serum was prepared from the blood and a weighed amount dissolved in 2 ml 1 M NaOH/0.2% SDS. The peak height of the fluorescence emission (usually between 658 and 664 nm) was measured with a fluorometer (Fluorolog 2, Spex Industries, Edison, NJ) (excitation at 400 nm, emission scanned from 580–720 nm). Quantitation of $c_{e6}$ concentration in the tissue extracts was obtained by constructing calibration curves from known amounts of the same conjugate together with specific tissue samples from uninjected mice dissolved in 1 M NaOH/0.2% SDS. A separate calibration curve was constructed for each combination of PS and each tissue type. In agreement with previous reports (Weagle et al, 1988) we found endogenous chlorin-like fluorescence emission spectra in tissue extracts from stomach and intestines and to a much lesser extent in the skin of non-injected mice. These values were variable and generally lower than that delivered to these organs by injected $c_{e6}$ derivatives, and the mean values of endogenous fluorescence per gram tissue from 9 control mice were subtracted from the values found in skin, stomach and intestine tissue in the mice injected with $c_{e6}$ conjugates before conversion to pmol $c_{e6}$ equivalent.

### Histology

During necropsy mice were carefully examined in the entire abdominal cavity. Pieces of tissue (200–300 mg) were removed and immediately placed in 10% formalin followed by embedding in paraffin. Sections were cut 5 µm thick and stained with haematoxylin and eosin.

### Statistics

Differences between two means were assessed for significance by the two-tailed Student's *t*-test assuming equal or unequal variances of the standard deviations as appropriate. Standard errors of the ratios of two means were calculated in quadrature.

### RESULTS

### Tumour model

After the injection of $5 \times 10^6$ HT-29 cells into the liver, mice were inspected for the establishment and evaluation of hepatic tumours.

 *© 2000 Cancer Research Campaign*

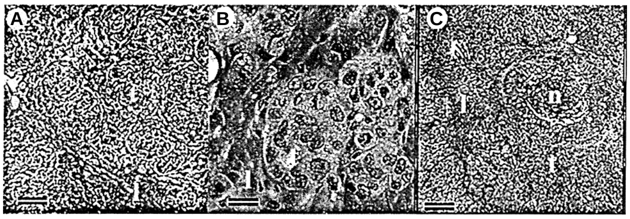

**Figure 2** Haematoxylin and eosin stained fixed and paraffin embedded histological sections from left lateral lobes of liver bearing HT 29 tumours. (**A**) Section of tumour from mouse 9 days after implantation; 1 = normal liver, t = tumour, scale bar = 100 μm. (**B**) Section of tumour from mouse 9 days after implantation, scale bar = 25 μm. (**C**) Section of tumour from mouse 18 days after implantation, n = necrosis, scale bar = 200 μm

They were examined at laparotomy 3, 9, 18 and 40 days after tumour injection. At the third day no tumour was visible in the lobe, but a small mass was detectable by palpitation of the lobe with forceps. Within 9 days of injection all the mice had visible hepatic tumour localized in the lobe injected and the diameter ranged from 5–7 mm. Macroscopically, the tumour appeared irregular and the lobe surface was grey-white and sometimes appeared umbilicated. There was a distinct border between the tumour and the normal liver tissue, in some cases small satellite nodules were found close to the tumour (Figure 2a). Microscopically, the histology was moderately to poorly differentiated adenocarcinoma from colorectal cancer (Figure 2b). At this time there was only a small area of necrosis within the tumour. The uninjected lobe of the liver, colon, spleen, lung, stomach and kidney were grossly and histologically examined and no evidence of metastases was found. 18 days after injection the tumour involved the entire left lateral lobe and also the principal lobe, about half of the liver was completely involved with tumour and appeared bigger, irregular grey-white, with areas of necrosis and dilated vessels on the surface, microscopically a more significant area of central necrosis was found (Figure 2c). Tumour weight varied from 0.9–1.2 g and remaining normal liver from 0.8–1.6 g (control mouse livers ranged from 1.5–2.0 g). At 40 days after implantation the tumour involved the whole liver. The shape of the lobes was conserved but with bigger dimensions; the weight of the tumour was 2.8–3.3 g and remaining normal liver 0.3–0.7 g. At 40 days ascites was found in all the mice and macroscopically the tumour was grey-red, necrotic and bleeding, microscopically a lot of necrotic areas were found and all the normal parenchyma was substituted by tumour. Mice were sacrificed at the 40-day time-point to avoid undue suffering. This animal model produces a single intrahepatic metastasis of colorectal cancer suitable for insertion of a diffuser-tipped fibreoptic for interstitial illumination.

### Biodistribution

The method for extraction of the $c_{e6}$ from tissue samples and serum has been shown to give reliable results in a previous publication (Duska et al, 1997). The values for the content of $c_{e6}$ expressed in pmol $g^{-1}$ tissue extracted from the different organs when mice were sacrificed 3 h after injection are presented in Table 1. The corresponding values for percentage injected dose $c_{e6}$ per gram of tissue, and tumour-to-normal liver and tumour-to-skin ratios are shown in Table 2. It can readily be seen that the tumour and the normal liver had high levels of PS compared to other organs for all 5 compounds investigated. The absolute amount of $c_{e6}$ delivered to the tumour by the anionic 17.1A-pl-$c_{e6}$-succ (1044 ± 207 pmol $g^{-1}$) was significantly higher than any other tissue or other compound. Both the anionic species (17.1A-pl-$c_{e6}$-succ and pl-$c_{e6}$-succ) had similar accumulations in normal liver, and for the non-antibody targeted pl-$c_{e6}$-succ this was similar to the value in the tumour, while for the Mab targeted 17.1A-pl-$c_{e6}$-succ the tumour had almost twice as much. Both cationic species delivered significantly smaller amounts of $c_{e6}$ to both tumour and normal liver than their anionic counterparts. The low values remaining in the blood after injection of both cationic species were balanced by the high values in the lungs, suggesting that the lungs relatively quickly take up both the cationic species. The bladder also had a high level of $c_{e6}$ for all 5 compounds, while the level in the skin was very low for cationic compounds, low for anionic compounds, and high for free $c_{e6}$. The spleen had remarkably low levels of $c_{e6}$ delivered by all 5 compounds, as did the muscle and heart. Tumour-to-normal liver ratios were significantly greater than one for PICs of both charges, the cationic pl-$c_{e6}$ and free $c_{e6}$. Although the tumour-to-normal liver ratio was highest for free $c_{e6}$, this appeared to be a function of a low uptake in liver, rather than a high affinity for tumour. Tumour-to-skin ratios were very high for all the conjugates (≥ 38), compared to that of free $c_{e6}$ (1.94). It should be noted that the mean of the tumour-to-normal tissue ratios is not

**Table 1** Biodistribution of $c_{e6}$ in tissues at 3 hours post-injection

| | **17.1A-pl-$c_{e6}$-succ ($n$ = 10)** | **17.1A-pl-$c_{e6}$ ($n$ = 7)** | **pl-$c_{e6}$-succ ($n$ = 8)** | **pl-$c_{e6}$ ($n$ = 7)** | **free $c_{e6}$ ($n$ = 6)** |
|---|---|---|---|---|---|
| Liver | 520 ± 106 | 102 ± 24 | 560 ± 148 | 209 ± 73 | 92 ± 19 |
| Tumour | 1044 ± 207 | 188 ± 37 | 441 ± 181 | 373 ± 123 | 313 ± 54 |
| Blood | 217 ± 101 | 29 ± 14 | 263 ± 42 | 43 ± 41 | 517 ± 260 |
| Skin | 108 ± 98 | 10 ± 11 | 127 ± 69 | 16 ± 16 | 347 ± 199 |
| Muscle | 11 ± 13 | 18 ± 9 | 39 ± 17 | 6 ± 7 | 55 ± 36 |
| Kidney | 216 ± 70 | 32 ± 7 | 74 ± 31 | 39 ± 7 | 28 ± 7 |
| Spleen | 8 ± 7 | 3 ± 2 | 4 ± 3 | 14 ± 6 | 12 ± 5 |
| Bladder | 107 ± 69 | 225 ± 59 | 162 ± 79 | 162 ± 85 | 295 ± 187 |
| Lung | 27 ± 17 | 573 ± 200 | 16 ± 6 | 539 ± 149 | 7 ± 3 |
| Heart | 14 ± 8 | 4 ± 2 | 9 ± 9 | 2 ± 2 | 2 ± 1 |
| Small bowel | 87 ± 68 | 65 ± 44 | 44 ± 42 | 107 ± 66 | 189 ± 80 |
| Stomach | 94 ± 84 | 34 ± 29 | 51 ± 51 | 57 ± 19 | 57 ± 43 |

Mice were sacrificed 3 h after i.v. injection of 0.25 mg $c_{e6}$ equivalent/kg body weight. Samples of tumour and normal organs (≈ 100 mg) were extracted, the fluorescence measured and converted to pmol $c_{e6}$ equivalent by comparison with standard fluorescence curves of known amounts of conjugate dissolved with individual tissue samples. Fluorescence values from tissue taken uninjected mice were subtracted to correct for autofluorescence. All values are expressed in pmol $c_{e6}$ equivalent per g wet weight of tissue, and errors are SEM.

*© 2000 Cancer Research Campaign* 

**Table 2** Tissue $c_{e6}$ content (% ID/g tissue) and mean of tumour-to-normal tissue ratios at 3 h

| | Tumour (% ID/g)[a] | *P* vs PIC-[b] | Liver (% ID/g) | Tumour/liver[c] | Tumour/skin |
|---|---|---|---|---|---|
| 17.1A-pl-$c_{e6}$-succ | 12.58 ± 2.49% | | 6.27 ± 1.28% | 2.52 ± 0.71 | 68.5 ± 15.4 |
| 17.1A-pl-$c_{e6}$ | 2.27 ± 0.45% | 0.003 | 1.23 ± 0.29% | 2.2 ± 0.45 | 71.7 ± 19.7 |
| pl-$c_{e6}$-succ | 5.31 ± 2.18% | 0.034 | 6.75 ± 1.78% | 0.73 ± 0.17 | 38.0 ± 19.4 |
| pl-$c_{e6}$ | 4.49 ± 1.48% | 0.019 | 2.52 ± 0.88% | 1.98 ± 0.38 | 81.4 ± 14.2 |
| free $c_{e6}$ | 3.77 ± 0.65% | 0.024 | 1.11 ± 0.23% | 3.61 ± 0.39 | 1.94 ± 0.9 |

[a]Mean tumour content of $c_{e6}$ equivalent per gram tissue as a percentage of total injected dose per mouse ± SEM. [b]Unpaired two-tailed Student's *t*-test was used to compare the values of tumour %ID/g for other conjugates versus the value determined for 17.1A-pl-$c_{e6}$-succ (12.58 ± 2.49%). [c]Mean of individual tumour-to-normal liver ratios derived from each mouse ± SEM.

**Table 3** Biodistribution of $c_{e6}$ in tissues at 24 hours post-injection

| | 17.1A-pl-$c_{e6}$-succ (*n* = 7) | 17.1A-pl-$c_{e6}$ (*n* = 6) | pl-$c_{e6}$-succ (*n* = 7) | pl-$c_{e6}$ (*n* = 6) | free $c_{e6}$ (*n* = 6) |
|---|---|---|---|---|---|
| Liver | 452 ± 153 | 59 ± 15 | 353 ± 101 | 164 ± 47 | 30 ± 1 |
| Tumour | 664 ± 177 | 89 ± 36 | 418 ± 246 | 216 ± 113 | 41 ± 23 |
| Blood | 2 ± 1 | 4 ± 3 | 24 ± 12 | 14 ± 10 | 25 ± 15 |
| Skin | 15 ± 16 | 0 | 75 ± 28 | 27 ± 20 | 20 ± 17 |
| Muscle | 5 ± 5 | 8 ± 9 | 9 ± 6 | 0 | 23 ± 25 |
| Kidney | 41 ± 8 | 8 ± 3 | 32 ± 9 | 14 ± 5 | 5 ± 1 |
| Spleen | 17 ± 9 | 26 ± 17 | 81 ± 20 | 23 ± 7 | 4 ± 2 |
| Bladder | 128 ± 69 | 18 ± 9 | 255 ± 75 | 94 ± 65 | 55 ± 6 |
| Lung | 12 ± 3 | 103 ± 46 | 8 ± 2 | 315 ± 124 | 3 ± 1 |
| Heart | 8 ± 4 | 0 | 3 ± 2 | 2 ± 1 | 0 |
| Small bowel | 95 ± 37 | 33 ± 26 | 89 ± 63 | 202 ± 142 | 16 ± 12 |
| Stomach | 77 ± 66 | 64 ± 61 | 107 ± 61 | 12 ± 13 | 19 ± 20 |

See caption to Table 1.

**Table 4** Tissue $c_{e6}$ content (% ID/g tissue) and mean of tumour-to-normal tissue ratios at 24 h

| | Tumour (% ID/g)[a] | P vs PIC-[b] | Liver (%ID/g) | Tumour/liver[c] | Tumour/skin |
|---|---|---|---|---|---|
| 17.1A-pl-$c_{e6}$-succ | 8.00 ± 2.13% | | 5.45 ± 1.84% | 1.54 ± 0.4 | 87.1 ± 13.9 |
| 17.1A-pl-$c_{e6}$ | 1.07 ± 0.43% | 0.011 | 0.71 ± 0.18% | 1.6 ± 0.59 | 52.0 ± 24.5 |
| pl-$c_{e6}$-succ | 5.04 ± 2.96% | n.s. | 4.25 ± 1.22% | 1.7 ± 1.17 | 27.5 ± 14.0 |
| pl-$c_{e6}$ | 2.60 ± 1.36% | 0.043 | 1.98 ± 0.57% | 1.7 ± 0.8 | 59.7 ± 20.5 |
| free $c_{e6}$ | 0.49 ± .28% | 0.009 | 0.36 ± 0.1% | 2.6 ± 1.73 | 2.16 ± 24.1 |

See caption to Table 2.

necessarily numerically equal to the ratio of the mean $c_{e6}$ contents in tumour and normal tissue as can be seen by comparing the values in Table 2 with those calculated from Table 1.

The corresponding values for $c_{e6}$ content of various tissue samples and ratios, at 24 hours after injection, are given in Tables 3 and 4. Again the absolute amount of $c_{e6}$ in the tumour delivered by the anionic PIC was greater than any other tissue or compound, but the difference between this value and that delivered to tumour by pl-$c_{e6}$-succ was no longer significant. The absolute amounts of $c_{e6}$ that were retained in both tumour and liver were higher for both anionic species. Considerable amounts of $c_{e6}$ were retained in the lungs when delivered by cationic species, and the values in blood were uniformly low for all the compounds. Free $c_{e6}$ appeared to have been almost totally eliminated from all the organs of the mice by 24 hours. The tumour-to-normal liver ratios were lower at 24 h than at 3 h for all compounds except pl-$c_{e6}$-succ. Again tumour-to-skin ratios were high for all the conjugates but not free $c_{e6}$.

Figure 3 presents the values for the $c_{e6}$ content remaining in tumour and normal liver at 24 hours expressed as a percentage of that measured at 3 hours. It can be seen that the amounts of free $c_{e6}$

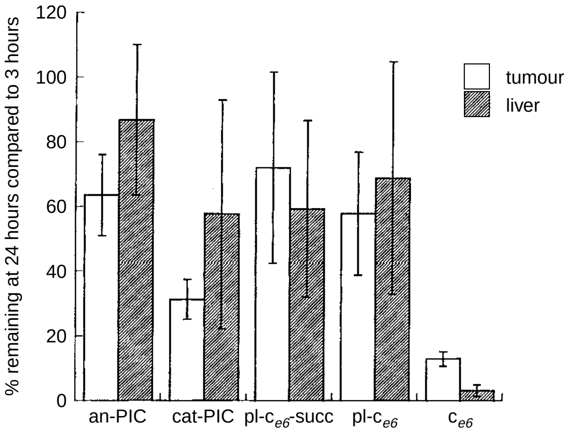

**Figure 3** Pharmacokinetics of conjugates and free $c_{e6}$ in tumour and normal liver. Values of mean $c_{e6}$ equivalent content in tumour and normal liver at 24 h as a fraction of the corresponding mean values at 3 h ± SEM calculated in quadrature

 *© 2000 Cancer Research Campaign*

remaining in both tumour (13%) and liver (3%) were very low compared to those found with the conjugates. The value found for the cationic PIC remaining in the tumour (31%) is significantly lower compared to that found for the anionic PIC (64%, $P < 0.05$). All the other values are relatively similar at between 55 and 85% remaining and there are no significant differences between them.

### DISCUSSION

Optimizing PDT involves attempting to increase both the absolute tumour content of PS and the selectivity between tumour and surrounding normal tissue. For many tumour types selectivity may be provided by spatial confinement of illumination, but for tumours in more complex sites such as the liver or diffuse intraperitoneal carcinomatosis (Duska et al, 1997) this may not be possible. Hepatic metastases of colorectal cancer are only rarely thought to be eligible for surgical resection due to the common occurrence of mulifocality, involvement of major blood vessels, and extrahepatic disease (Steele and Ravikumar, 1989). Although PDT has been suggested for treatment of liver metastases (van Hillegersberg et al, 1992), it has not found much support due to the relative lack of selectivity for tumour as opposed to normal liver (which accumulates large amounts of clinically employed PS such as Photofrin® (Van Hillegersberg et al, 1992)). Mab conjugates have been proposed as targeting vehicles to increase the selectivity of PS for tumours (Hasan, 1992; Yarmush et al, 1993; Klyashchitsky et al, 1994). It was attractive to explore the ability of the Mab 17.1A that is in clinical use for treating liver metastases of colorectal cancer (Nieroda et al, 1991; Riethmuller et al, 1994) to target a PS to tumour cells while sparing normal liver.

We have previously shown (Hamblin et al, 1996; Duska et al, 1997) that poly-L-lysine can be used as a linker to attach several $c_{e6}$ molecules to a Mab in a site-specific manner which preserves as much as possible the antigen recognizing site of the Mab. In addition this approach allows the preparation of these PICs with either polycationic or polyanionic charges. In a previous report (Del Governatore et al, 2000a) PICs prepared from the Mab 17.1A were tested for cellular uptake, localization, specificity and phototoxicity against HT29 cells in vitro. However we had not previously compared the delivery of PICs of opposite charge to tumours after i.v. administration.

The results from the present biodistribution study showed that both the presence of the tumour-targeting Mab, and the overall charge borne by the conjugate had significant effects on both the absolute amount of PS in the tumour, and on the selectivity for tumour over normal liver. The charge borne by the conjugates clearly affected their biological processing. Cationic charge led to very high uptake in the lungs, and relatively low levels in blood and other organs. This finding is in agreement with a report (Ekrami et al, 1995), which investigated the biodistribution of Bowman-Birk protease inhibitor conjugated to various polylysines. These investigators found that the accumulation in the lungs correlated well with the size and degree of polycationic charge of their conjugates. Pardridge et al (1998) found that giving a Mab a polycationic charge reduced its serum half-life in rats after i.v. administration to less than 5% of the unmodified level. The present study found that the uptake of both the antibody-targeted and non-antibody-targeted polyanionic conjugates was very much higher than their polycationic counterparts in both tumour and normal liver. This difference may be partly explained by the fact that each anionic 17.1A conjugate had twice the loading of $c_{e6}$ compared to each cationic PIC, but even accounting for this difference, the anionic PIC was still much more efficient in delivering $c_{e6}$ to the tumour. The amount of $c_{e6}$ delivered by the anionic PIC expressed as % injected dose $g^{-1}$ was 12.5, which compares well with values delivered to mouse xenograft tumours by radiolabelled Mabs (Duewell et al, 1986; Sato et al, 1999). This difference between anionic and cationic PICs administered i.v. is in clear contrast with a study from our laboratory (Duska et al, 1997) in which polycationic and polyanionic PICs were constructed in a similar fashion to the present PICs, but conjugated to the Mab OC125 which was targeted against human ovarian cancer growing as disseminated intraperitoneal tumours in nude mice. After i.p. injection it was found that the cationic PIC delivered several times more $c_{e6}$ to the tumour than the anionic counterpart, both at 3 h and 24 h after injection. The ratio of the $c_{e6}$ content in the i.p. tumour to that in the intestines (the critical organ for i.p. delivery) was also higher for the cationic PIC. The findings from these two studies led to the hypothesis that polyanionic conjugates perform better after i.v. delivery, while polycationic conjugates perform better after intracavitary delivery. It is possible that administration of the PIC via the hepatic artery could give even better selectivity for the tumour over normal liver (Rougier, 1998). Nishiwaki et al (1989) used intra-arterial Lipiodol (a contrast medium composed of iodized castor oil) to transport the PS pheophorbide a to VX-2 liver tumours in rabbits, and found tumour-to-normal liver ratios > 30 times higher than those found when an i.v. injection of water soluble PS was used. Rovers et al (1999) found that hepatic artery administration of the PS Foscan gave significantly higher levels of PS in the liver tumour and better selectivity over normal liver than femoral vein administration.

Does the presence of the tumour-targeting Mab lead to increased specificity for the tumour compared to normal liver? In the case of the anionic PIC at 3 h there was a significant increase in the tumour-to-normal liver ratio, 2.52 compared to 0.73 ($P$ = 0.028) found with the non-antibody-targeted pl-$c_{e6}$-succ, but this differences disappears at 24 h. The cationic PIC and the cationic pl-$c_{e6}$ did not show any significant differences in tumour-to-normal liver ratios either at 3 h or 24 h, but the values at 3 h were both significantly greater than one. These latter findings may be explained by a report (Kornguth et al, 1991) that cationic macromolecules may possess a tumour-localizing ability independent of any antibody interaction, and in a study of the biodistribution of radiolabelled polylysines in orthotopic C6 gliomas growing in rats they found tumour-to-normal brain ratios of up to 10. Tumour selectivity was lost when the polymers were rendered polyanionic by complete succinylation. These authors attributed (Kornguth et al, 1989) the tumour localizing ability of polycationic macromolecules to binding to polysialic acid residues overexpressed on the membrane of cancer cells.

Free $c_{e6}$ gave only very low tissue uptakes although the selectivity for the tumour was quite good. This is in agreement with a report (Orenstein et al, 1996) which found that $c_{e6}$ had higher selectivity for tumours than the other PS studied. It has been well-established that the main side effect of clinical PDT is cutaneous photosensitivity, which may entail a patient keeping out of direct light for some time (Tralau et al, 1989; Baas et al, 1995). For this reason it is of interest to study the uptake of PS in the skin in addition to the tumour and normal liver. In Table 2 it can be seen that the tumour-to-skin ratios obtained with all the conjugates were very high compared to that found with free $c_{e6}$, suggesting that

© 2000 Cancer Research Campaign 

macromolecular delivery might decrease cutaneous photosensitivity after PDT.

In conclusion we have shown that the PIC with a polyanionic charge delivers both higher absolute amounts of $c_{e6}$ to the tumour, and gives higher tumour:normal liver ratios, than the PIC with a polycationic charge. Both the pharmacokinetics and biodistribution of conjugated $c_{e6}$ are very different from free $c_{e6}$, regardless of targeted binding. Based on the data in the current study, a recent report (Del Governatore et al, 2000b) will describe the use of experimental photoimmunotherapy in this model. The anionic PIC gave significant reductions in tumor weight and increased survival of the mice not seen with free $c_{e6}$. Photoimmunotherapy might also be applied to other liver tumours for which Mabs are available, such as primary hepatocellular carcinomas, and metastases from other primaries such as melanoma, breast and ovarian cancer.

### ACKNOWLEDGEMENTS

We thank Centacor Inc for the gift of 17.1A Mab. This work was funded by the NIH (Grant R01 AR40352) and the Department of Defense Medical Free Electron Laser program (Grant N 00014-94-1-0927).

### REFERENCES

Baas P, van Mansom I, van Tinteren H, Stewart FA and van Zandwijk N (1995) Effect of N-acetylcysteine on Photofrin-induced skin photosensitivity in patients. *Lasers Surg Med* **16**: 359–367

Del Governatore M, Hamblin MR, Piccinini EE, Ugolini G and Hasan T (2000a) Targeted photodestruction of human colon cancer cells using charged 17.1A chlorin e6 immunoconjugates. *Br J Cancer* **82**: 56–64

Del Governatore M, Hamblin MR, Shea CR, Rizvi I, Molpus KL, Tanabe K and Hasan T (2000b) Experimental photoimmunotherapy of hepatic metastases of colorectal cancer with a 17.1 A chlorin*e6* immunoconjugate. *Cancer Res* **60**: 4200–4205

Dougherty TJ, Gomer CJ, Henderson BW, Jori G, Kessel D, Korbelik M, Moan J and Peng Q (1998) Photodynamic therapy. *J Natl Cancer Inst* **90**: 889–905

Duewell S, Horst W and Westera G (1986) Uptake of a monoclonal antibody against CEA (Tumak 431/31) in a human colon tumor (Co-112) xenografted in the nude mouse. Dependence on tumor size and injected dose. *Cancer Immunol Immunother* **23**: 101–106

Duska LR, Hamblin MR, Bamberg MP and Hasan T (1997) Biodistribution of charged F(ab´)2 photoimmunoconjugates in a xenograft model of ovarian cancer. *Br J Cancer* **75**: 837–844

Ekrami H, Kennedy AR and Shen WC (1995) Disposition of positively charged Bowman-Birk protease inhibitor conjugates in mice: influence of protein conjugate charge density and size on lung targeting. *J Pharm Sci* **84**: 456–461

Goff BA, Bamberg MP and Hasan T (1992) Experimental photodynamic treatment of ovarian cancer cells with immunoconjugates. *Antibody Immunocon Radiopharm* **5**: 191–199

Goff BA, Hermanto U, Rumbaugh J, Blake J, Bamberg M and Hasan T (1994) Photoimmunotherapy and biodistribution with an OC125-chlorin immunoconjugate in an in vivo murine ovarian cancer model. *Br J Cancer* **70**: 474–480

Goff BA, Blake J, Bamberg MP and Hasan T (1996) Treatment of ovarian cancer with photodynamic therapy and immunconjugates in a murine ovarian cancer model. *Br J Cancer* **74**: 1194–1198

Gottlinger HG, Funke I, Johnson JP, Gokel JM and Riethmuller G (1986) The epithelial cell surface antigen 17-1A, a target for antibody-mediated tumor therapy: its biochemical nature, tissue distribution and recognition by different monoclonal antibodies. *Int J Cancer* **38**: 47–53

Hamblin MR, Miller JL and Hasan T (1996) The effect of charge on the interaction of site-specific photoimmunoconjugates with human ovarian cancer cells. *Cancer Res* **56**: 5205–5210

Hasan T (1992) Photosensitizer delivery mediated by macromolecular carrier systems. In: Henderson B and Dougherty T (eds) *Photodynamic Therapy: Basic Principles and Clinical Applications.* Marcel Dekker: New York, NY, pp. 187–200

Klyashchitsky BA, Nechaeva IS and Ponomaryov GV (1994) Approaches to targetted photodynamic therapy. *J Control Release* **29**: 1–16

Kornguth SE, Kalinke T, Robins HI, Cohen JD and Turski P (1989) Preferential binding of radiolabeled poly-L-lysines to C6 and U87 MG glioblastomas compared with endothelial cells in vitro. *Cancer Res* **49**: 6390–6395

Kornguth S, Lohmiller S, Kalinke T, Robins HI, Kyritsis A, Turski P, Cohen J, Nickles RJ and DeJesus O (1991) Biodistribution of polylysine-guided radionuclides. In: Epenetos A (ed) *Monoclonal Antibodies.* Chapman & Hall Medical: London, pp. 133–147

Litvinov SV, Velders MP, Bakker HA, Fleuren GJ and Warnaar SO (1994) Ep-CAM: a human epithelial antigen is a homophilic cell-cell adhesion molecule. *J Cell Biol* **125**: 437–446

Meredith RF, Khazaeli MB, Plott WE, Spencer SA, Wheeler RH, Brady LW, Woo DV and LoBuglio AF (1995) Initial clinical evaluation of iodine-125-labeled chimeric 17-1A for metastatic colon cancer. *J Nucl Med* **36**: 2229–2233

Mew D, Wat CK, Towers GH and Levy JG (1983) Photoimmunotherapy: treatment of animal tumors with tumor-specific monoclonal antibody-hematoporphyrin conjugates. *J Immunol* **130**: 1473–1477

Nieroda CA, Mojzisik C, Hinkle G, Thurston MO and Martin EW, Jr (1991) Radioimmunoguided surgery (RIGS) in recurrent colorectal cancer. *Cancer Detect Prev* **15**: 225–229

Nishiwaki Y, Nakamura S and Sakaguchi S (1989) New method of photosensitizer accumulation for photodynamic therapy in an experimental liver tumor. *Lasers Surg Med* **9**: 254–263

Orenstein A, Kostenich G, Roitman L, Shechtman Y, Kopolovic Y, Ehrenberg B and Malik Z (1996) A comparative study of tissue distribution and photodynamic therapy selectivity of chlorin e6, Photofrin II and ALA-induced protoporphyrin IX in a colon carcinoma model. *Br J Cancer* **73**: 937–944

Pardridge WM, Buciak J, Yang J and Wu D (1998) Enhanced endocytosis in cultured human breast carcinoma cells and in vivo biodistribution in rats of a humanized monoclonal antibody after cationization of the protein. *J Pharmacol Exp Ther* **286**: 548–554

Riethmuller G, Schneider-Gadicke E, Schlimok G, Schmiegel W, Raab R, Hoffken K, Gruber R, Pichlmaier H, Hirche H and Pichlmayr R (1994) Randomised trial of monoclonal antibody for adjuvant therapy of resected Dukes' C colorectal carcinoma. German Cancer Aid 17-1A Study Group. *Lancet* **343**: 1177–1183

Rougier P (1998) Are there indications for intraarterial hepatic chemotherapy or isolated liver perfusion? The case of liver metastases from colorectal cancer. *Recent Results Cancer Res* **147**: 3–12

Rovers JP, Saarnak AE, Sterenborg HJ, Grahn MF and Terpstra OT (1999) Increased selectivity and uptake of Foscan (mTHPC) in a rat liver tumor model by hepatic artery administration. (Abstract). In: *European Society for Photobiology*. Granada, Spain, pp. 123

Sato N, Saga T, Sakahara H, Yao Z, Nakamoto Y, Zhang M, Kuroki M, Matsuoka Y, Iida Y and Konishi J (1999) Intratumoral distribution of radiolabeled antibody and radioimmunotherapy in experimental liver metastases model of nude mouse. *J Nucl Med* **40**: 685–692

Schmidt S, Wagner U, Oehr P and Krebs D (1992a) Clinical use of photodynamic therapy in gynecologic tumor patients – antibody-targeted photodynamic laser therapy as a new oncologic treatment procedure. *Zentralbl Gynakol* **114**: 307–311

Schmidt S, Wagner U, Schultes B, Oehr P, Decleer W, Ertmer W, Lubaschowski H, Biersack HJ and Krebs D (1992b) Photodynamic laser therapy with antibody-bound dyes. A new procedure in therapy of gynecologic malignancies. *Fortschr Med* **110**: 298–301

Steele G, Jr and Ravikumar TS (1989) Resection of hepatic metastases from colorectal cancer. Biologic perspective. *Ann Surg* **210**: 127–138

Tralau CJ, Young AR, Walker NP, Vernon DI, MacRobert AJ, Brown SB and Bown SG (1989) Mouse skin photosensitivity with dihaematoporphyrin ether (DHE) and aluminium sulphonated phthalocyanine (AlSPc): a comparative study. *Photochem Photobiol* **49**: 305–312

Van Cutsem E (1996) A glimpse of the future, new directions in the treatment of colorectal cancer. *Eur J Cancer* **32AS**: 23–27

van Hillegersberg R, Marijnissen JP, Kort WJ, Zondervan PE, Terpstra OT and Star WM (1992a) Interstitial photodynamic therapy in a rat liver metastasis model. *Br J Cancer* **66**: 1005–1014

Van Hillegersberg R, Van den Berg JW, Kort WJ, Terpstra OT and Wilson JH (1992b) Selective accumulation of endogenously produced porphyrins in a liver metastasis model in rats. *Gastroenterology* **103**: 647–651

 *© 2000 Cancer Research Campaign*

van Hillegersberg R, Pickering JW, Aalders M and Beek JF (1993) Optical properties of rat liver and tumor at 633 nm and 1064 nm: photofrin enhances scattering. *Lasers Surg Med* **13**: 31–39

Weagle G, Paterson PE, Kennedy J and Pottier R (1988) The nature of the chromophore responsible for naturally occurring fluorescence in mouse skin. *J Photochem Photobiol B* **2**: 313–320

Woodburn KW, Stylli S, Hill JS, Kaye AH, Reiss JA and Phillips DR (1992) Evaluation of tumour and tissue distribution of porphyrins for use in photodynamic therapy. *Br J Cancer* **65**: 321–328

Yarmush ML, Thorpe WP, Strong L, Rakestraw SL, Toner M and Tompkins RG (1993) Antibody targeted photolysis. *Crit Rev Ther Drug Carrier Syst* **10**: 197–252

© 2000 Cancer Research Campaign
